# Expansion and scale-up of HIV care and treatment services in four countries over ten years

**DOI:** 10.1371/journal.pone.0231667

**Published:** 2020-04-16

**Authors:** Chloe A. Teasdale, Elaine J. Abrams, Katharine A. Yuengling, Matthew R. Lamb, Chunhui Wang, Mirriah Vitale, Mark Hawken, Zenebe Melaku, Harriet Nuwagaba-Biribonwoha, Wafaa M. El-Sadr

**Affiliations:** 1 Department of Epidemiology & Biostatistics, CUNY Graduate School of Public Health and Health Policy, New York, NY, United States of America; 2 ICAP-Columbia University, Mailman School of Public Health, Columbia University, New York, NY, United States of America; 3 Department of Epidemiology, Mailman School of Public Health Columbia University, New York, NY, United States of America; 4 Department of Pediatrics, Vagelos College of Physicians and Surgeons, Columbia University Irving Medical Center, New York, NY, United States of America; 5 ICAP-Columbia University, Maputo, Mozambique; 6 ICAP-Columbia University, Nairobi, Kenya; 7 ICAP-Columbia University, Addis Ababa, Ethiopia; 8 ICAP-Columbia University, Mbabane, Eswatini; 9 Vagelos College of Physicians and Surgeons, Columbia University Irving Medical Center, New York, NY, United States of America; University of Ghana College of Health Sciences, GHANA

## Abstract

**Background:**

Scale-up and expansion of antiretroviral therapy (ART) for people living with HIV (PLHIV) have been a global priority for more than 15 years.

**Methods:**

We describe PLHIV at enrollment in care and ART initiation in Ethiopia, Kenya, Mozambique and Tanzania from 2005–2014 and report on enrollment location, CD4 count and loss to follow-up (LTF), death, and combined attrition (LTF and death) pre- and post-ART initiation over time. Pre-ART outcomes were estimated using competing risk and post-ART using Kaplan-Meier estimators; LTF defined as no visit within six months pre-ART and 12 months after ART start.

**Results:**

From 2005–2014, 884,328 PLHIV enrolled in care at 350 health facilities, median age was 32.0 years (interquartile range [IQR] 26.0–42.0), and majority were female (66.5%). The proportion of PLHIV enrolled at primary and rural facilities increased from 12.9% and 15.3% in 2005–2006 to 43.5% and 41.7% in 2013–2014 (p<0.0001). Median CD4+ cell count at enrollment increased from 171 cell/mm^3^ in 2005–2006 (IQR 71–339) to 289 cell/mm^3^ in 2013–2014 (IQR 133–485) (p<0.0001). A total of 460,758 (57.4%) PLHIV initiated treatment. Cumulative risk of LTF for PLHIV prior to ART initiation 12 months after enrollment was 33.5% (95%CI 33.36–33.58) and 21.98% (95%CI 21.9–22.1) after ART initiation. Pregnant women and the youngest PLHIV group had the highest attrition after ART initiation, at 24 months 40.8% (95%CI 40.1–41.6) of pregnant women and 47.4% (95%CI 46.4–48.4) of PLHIV 15–19 years were not retained. Attrition at 12 months after enrollment among PLHIV regardless of ART status was 38.5% (95%CI 38.4–38.6).

**Conclusion:**

Over 10 years of HIV scale-up in four sub-Saharan African countries, close to a million PLHIV were enrolled in care increasingly at rural and primary facilities with increasing CD4 count. Loss to follow-up from HIV care remains alarmingly high, particularly among pregnant women and younger PLHIV.

## Background

Remarkable progress has been achieved in the scale-up of antiretroviral therapy (ART) and health services for people living with HIV (PLHIV) in resource limited settings (RLS). [1] In 2004, out of the estimated 38 million PLHIV worldwide, fewer than 400,000 in low and middle-income countries were on ART, only 7% of those eligible for treatment at the time. [2] As of mid-2019, among the estimated 37.9 million PLHIV, 24.5 million were on ART (64.6%), and there has been a 55% decline in AIDS deaths from the peak of 1.2 million in 2004 to 770,000 in 2018, largely due to the expansion of HIV care and antiretroviral treatment. [3]

The epicenter of the epidemic remains in sub-Saharan Africa (SSA), specifically Eastern and Southern Africa which are home to 54.4% of all PLHIV worldwide (20.6 million PLHIV). [3] Many countries in this region have made significant progress in expanding HIV testing and treatment services. Data from the Population-Based HIV Impact Assessments (PHIA) conducted in multiple African countries have highlighted the incredible success in HIV scale-up and progress towards the ambitious 2020 UNAIDS 90-90-90 targets for HIV testing, ART initiation, and viral load suppression. [4] In Eswatini, as one example, 87% of adult PLHIV identified through the household testing survey reported knowledge of their HIV status, 89% self-reported being on ART, and 91% were virally suppressed (<1,000 copies/mL). [5] Despite this progress, challenges persist in identifying, enrolling, and retaining all PLHIV in HIV care. Malawi, Zambia and Zimbabwe PHIA data showed that up to half of previously undiagnosed adults had CD4+ <350 cells/mm^3^ and that men were significantly more likely to have advanced disease at diagnosis. [6] UNAIDS and PHIA data also demonstrate persistent sex gaps in uptake of ART with 68% of women on treatment in 2018 compared to 55% of men. [3,7] In addition, HIV treatment programs continue to struggle to retain patients in follow-up. A recent meta-analysis of ART retention data found that only 65% of PLHIV starting ART in Africa were still on treatment after 36 months. [8] These findings underscore the ongoing challenges of continuing to expand HIV testing and treatment services while also striving to improve retention of PLHIV and achieve optimal outcomes.

We examined a large patient level dataset from 350 HIV care and treatment facilities in Ethiopia, Kenya, Mozambique, and Tanzania from 2005 through 2014 to describe the characteristics of PLHIV at entry to HIV care and at ART initiation over time. We also estimate loss to follow-up (LTF), mortality and combined attrition (LTF and death) over the ten year period. This analysis is one of the largest to date of PLHIV enrolling in routine HIV care and treatment services, and provides important insights into the changes over the initial decade of HIV treatment scale-up and the ongoing challenges faced by HIV programs across four severely affected countries.

## Methods

We examined routinely collected patient-level data from health facilities in Ethiopia, Kenya, Mozambique, and Tanzania as part of the Identifying Optimal Models of HIV Care in Africa study, funded by President’s Emergency Plan for AIDS Relief (PEPFAR) through the US Centers for Disease Control and Prevention (CDC). In partnership with the Ministries of Health in each country, all health facilities received support from ICAP at Columbia University and offered a standard package of services, including HIV testing, pre-ART and ART care, and prevention and treatment for opportunistic infections, per each country’s national guidelines. National ART eligibility guidelines in each country changed over the period of observation and are summarized in S1 Table. Ethics and administrative approvals were obtained from the Columbia University Medical Center institutional review board (IRB) and the Associate Director of Science Office at the CDC, as well as from review boards in each country.

The study population included all adult PLHIV ≥15 years of age who enrolled at the health facilities from January 1, 2005 through December 31, 2014. Patients who enrolled in care <12 months prior to the last date of data collection at each health facility were excluded. At all facilities, medical record data from routine care visits were entered into on-site electronic databases by trained data capturers with data quality support from ICAP. Loss to follow-up (LTF) was defined as not having a recorded outcome of death or transfer out and before ART initiation (pre-ART) as not having a clinic visit for >12 months and >6 months after ART initiation. Data on deaths and documented transfer to other facilities were ascertained from facility records. Time to LTF or death was calculated from the date of ART initiation to the date of death (if available) or the last visit date.

We describe characteristics of PLHIV at enrollment in HIV care and at time of ART initiation based on year of enrollment and country including age, sex, point of entry, CD4 cell count (CD4+), and WHO HIV disease stage (measured up to 90 days prior and 30 days after). Cochran-Armitage tests for proportions and Kruskal-Wallis tests for medians were used to compare characteristics of PLHIV at enrollment and ART initiation in the periods 2005–2006 and 2013–2014. We report cumulative incidence of LTF, death, and combined attrition (LTF and death) before (pre-ART) and following ART initiation among patients who were ART-naïve at enrollment (patients reporting prior ART or current ART at time of enrollment were excluded from retention analyses). We also analyzed LTF, death, and combined attrition for all enrolled patients regardless of ART status in order to measure overall lack of retention at enrollment facilities among all PLHIV entering care. Survival analyses were conducted using competing risk estimators for pre-ART outcomes (treating ART initiation as a competing risk for pre-ART death and combined attrition, and ART initiation and death as competing risks for pre-ART LTF). For outcomes after ART initiation and for the analysis of all enrolled patients, Kaplan-Meier estimators were used. Unadjusted sub-distributional hazards (pre-ART) and Cox proportional hazards models (following ART initiation) were used to compare cumulative incidence by groups. Statistical analyses were performed in SAS 9.3 and Stata 12.

## Results

**Fig 1:**
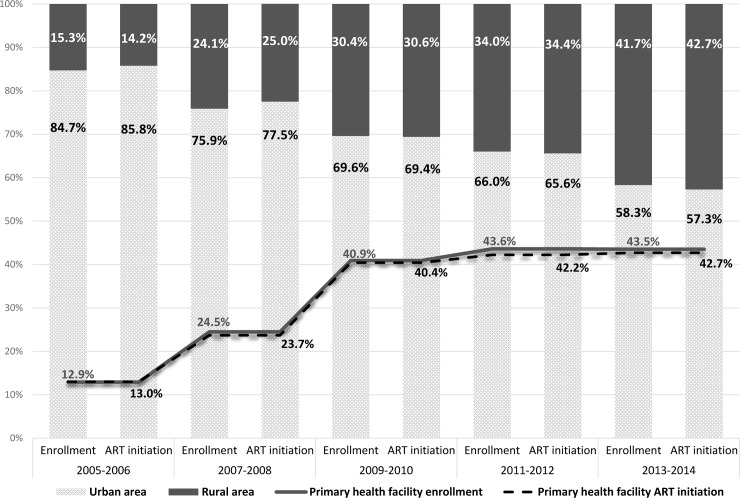
Proportions of PLHIV enrolling in HIV care and initiating ART by location (urban vs. rural) and at primary health facilities by year in Ethiopia, Kenya, Mozambique and Tanzania, 2005–2014 (N = 884,828).

**Fig 2:**
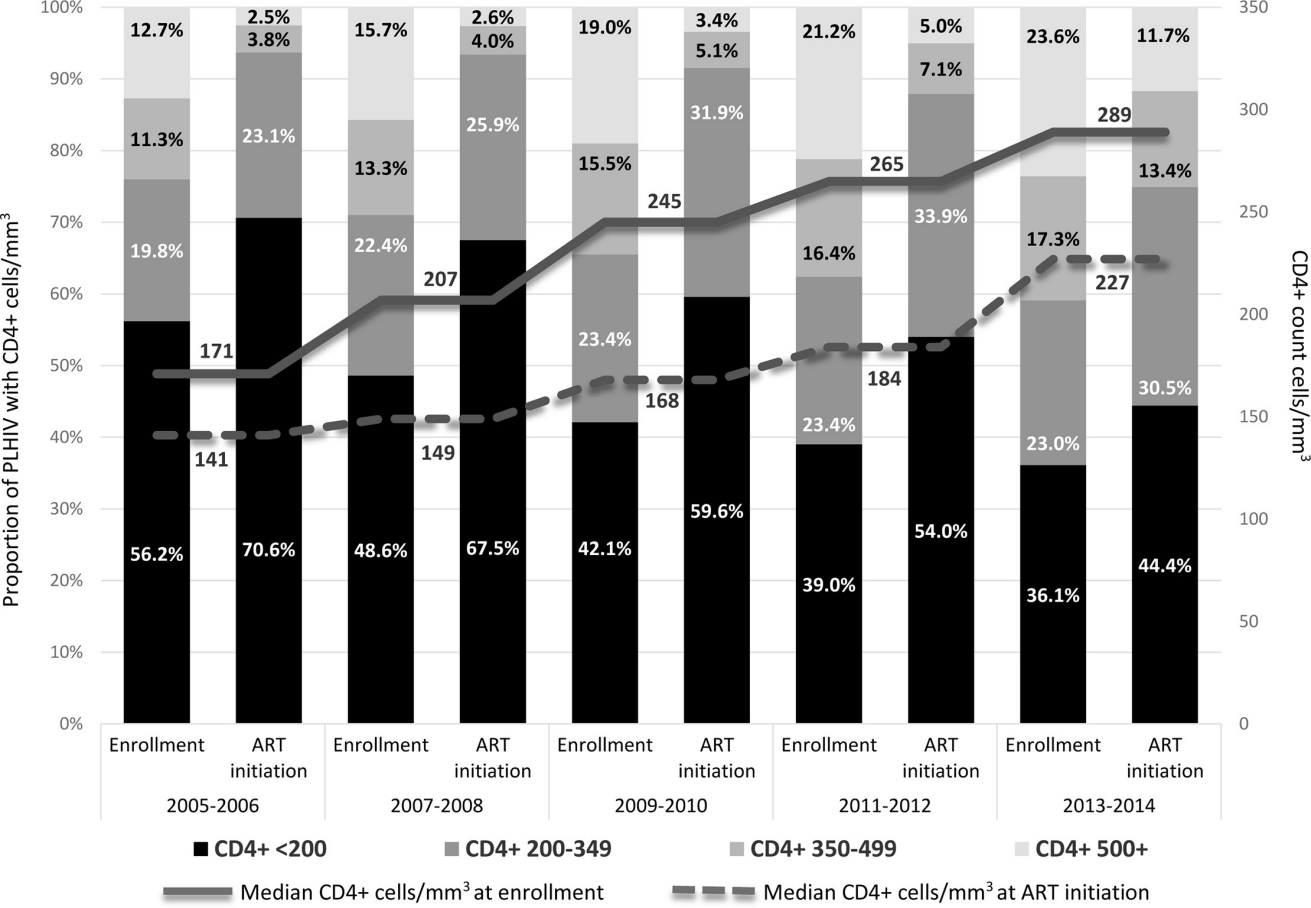
CD4+ count cell/mm^3^ at enrollment and ART initiation by category and median by year in Ethiopia, Kenya, Mozambique and Tanzania, 2005–2014 (N = 884,828).

A total of 884,328 patients enrolled in care at the 350 health facilities in the four countries between 2005 and 2014 (Table 1). The median duration of data collection at the individual facilities included in the analysis was 8 years (interquartile range (IQR) 7–9). Over time, more patients enrolled at primary health facilities; among all PLHIV 12.9% enrolled at primary health facilities in 2005–2006 compared to 43.5% in 2013–2014 (p<0.0001) (Fig 1). The proportion of PLHIV enrolled at rural health facilities also increased from 15.3% of patients in 2005–2006 to 41.7% in 2013–2014 (p<0.0001). The median age of all PLHIV at enrollment was 32.0 years (interquartile range [IQR] 26.0–42.0) with the proportion of patients 15–19 years increasing from 2.8% (2005–2006) to 6.5% (2013–2014) (p<0.0001). The majority were female (66.5%) and the proportion of those enrolled during a pregnancy increased from 4.8% in 2005–2006 to 25.0% in 2013-2014(p<0.0001) (Table 1). Median CD4+ at enrollment for all PLHIV increased from 171 cell/mm^3^ in 2005–2006 (IQR 71–339) to 289 cell/mm^3^ in 2013–2014 (IQR 133–485) (p<0.0001) (49.4% missing) (Fig 2). Among PLHIV with WHO stage at enrollment (79.9%), the proportion with WHO stage III or IV at enrollment decreased over time from 61.4% in 2005–2006 to 33.9% in 2013–2014 (p<0.0001) (Table 1). The proportion of PLHIV who were eligible for ART at entry to care according to prevailing national guidelines increased over time from 30.6% in 2005–2006 to 47.4% in 2013–2014 (p<0.0001). Overall, 18.2% of PLHIV had no visits recorded at the facility after the date of enrollment. S2 Table has characteristics of PLHIV at enrollment by country.

**Table 1:** Characteristics at enrollment in HIV care and at ART initiation among adults (> = 15 years) living with HIV enrolled in care at ICAP-supported facilities in Ethiopia, Kenya, Mozambique and Tanzania 2005–2014 (N = 884,328).

	All		Enrollment year									
			2005–2006		2007–2008		2009–2010		2011–2012		2013–2014	
	N	%	N	%	N	%	N	%	N	%	N	%
** **	884,328	100.0	122,034	13.8	231,309	26.2	238,038	26.9	185,251	20.9	107,696	12.2
**Country**												
Ethiopia	131,819	14.9	12,005	9.8	30,215	13.1	36,542	15.4	30,942	16.7	22,115	20.5
Kenya	365,809	41.4	48,850	40.0	89,090	38.5	92,717	39.0	85,569	46.2	49,583	46.0
Mozambique	234,648	26.5	39,261	32.2	64,634	27.9	67,444	28.3	39,578	21.4	23,731	22.0
Tanzania	152,052	17.2	21,918	18.0	47,370	20.5	41,335	17.4	29,162	15.7	12,267	11.4
**Number of health facilities reporting**	265		350		311		342		343		265	
**Age at enrolment, Median (IQR)**	32.0 (26.0–40.0)		34.0 (28.0–41.3)		32.3 (26.8–40.0)		32.2 (26.0–40.0)		31.5 (25.3–39.9)		30.8 (25.0–39.0)	
15–19 years	39,238	4.4	3,477	2.8	8,813	3.8	9,932	4.2	10,053	5.4	6,963	6.5
20–29 years	303,038	34.3	36,653	30.0	77,231	33.4	80,739	33.9	67,518	36.4	40,897	38.0
30–39 years	305,095	34.5	44,546	36.5	82,337	35.6	82,147	34.5	61,652	33.3	34,413	32.0
40–49 years	154,712	17.5	25,609	21.0	41,761	18.1	41,789	17.6	29,333	15.8	16,220	15.1
50+ years	82,245	9.3	11,749	9.6	21,167	9.2	23,431	9.8	16,695	9.0	9,203	8.5
**Male**	296,685	33.5	43,351	35.5	77,812	33.6	77,929	32.7	60,391	32.6	37,202	34.5
**Female**	587,643	66.5	78,683	64.5	153,497	66.4	160,109	67.3	124,860	67.4	70,494	65.5
Women pregnant at enrollment	66,970	16.7	2,141	4.8	11,789	12.5	17,543	16.0	21,100	22.5	14,397	25.0
**Point of entry**												
VCT	292,609	33.1	44,768	36.7	80,166	34.7	76,380	32.1	57,464	31.0	33,831	31.4
PMTCT	86,072	9.7	4,256	3.5	19,059	8.2	25,043	10.5	24,183	13.1	13,531	12.6
TB/HIV	21,463	2.4	3,643	3.0	7,134	3.1	5,117	2.1	3,645	2.0	1,924	1.8
Inpatient	42,245	4.8	5,481	4.5	13,935	6.0	13,428	5.6	6,941	3.7	2,460	2.3
Outpatient	123,888	14.0	11,902	9.8	29,864	12.9	33,280	14.0	28,206	15.2	20,636	19.2
Other	232,365	26.3	33,013	27.1	59,646	25.8	64,053	26.9	49,739	26.8	25,914	24.1
Unknown	85,686	9.7	18,971	15.5	21,505	9.3	20,737	8.7	15,073	8.1	9,400	8.7
**Transferred in**	55,533	14.0	4,006	7.6	11,989	12.1	19,758	17.1	12,350	15.8	7,430	14.9
**CD4+ enrollment**, median (IQR)	235 (103–426)		171 (71–339)		207 (92–388)		245 (111–434)		265 (121–459)		289 (133–485)	
CD4, women, median (IQR)	261 (120–460)		187 (80–364)		228 (105–417)		271 (130–468)		294 (140–492)		320 (156–521)	
CD4, men, median (IQR)	192 (80–362)		146 (59–291)		174 (72–335)		197 (82–365)		215 (90–387)		237 (103–412)	
**Missing CD4+ enrollment**	427,329	49.4	70,876	58.1	111,580	48.2	115,984	48.7	80,477	43.4	48,412	45.0
**WHO stage at enrollment**												
Stage I	220,165	31.2	15,750	18.4	45,646	25.6	62,435	32.0	58,601	38.0	37,733	40.6
Stage II	178,011	25.2	17,292	20.2	43,492	24.4	53,599	27.5	39,950	25.9	23,678	25.5
Stage III	254,036	36.0	43,169	50.5	72,811	40.8	65,826	33.7	45,876	29.7	26,354	28.3
Stage IV	54,043	7.7	9,300	10.9	16,332	9.2	13,337	6.8	9,853	6.4	5,221	5.6
**Missing WHO Stage at enrolment**	178,073	20.1	36,523	29.9	53,028	22.9	42,841	18.0	30,971	16.7	14,710	13.7
**Prior ART reported**	65,652	8.9	2,832	3.1	13,193	7.0	18,548	9.2	15,905	9.8	15,174	16.7
**On ART at time of enrollment**	16,328	3.4	1648	2.6	4280	3.5	4179	3.3	3672	3.8	2549	3.8
**Never returned after enrollment**	161,255	18.2	26,652	21.8	44,372	19.8	39,863	16.8	30,150	16.3	20,218	18.8
Never returned after enrollment & female	108,967	67.6	17,192	64.5	30,022	67.7	27,417	68.8	21,059	69.8	13,277	65.7
**PLHIV in pre-ART retention analyses**	807,118	91.3	122,034	100.0	230,746	99.8	235377	98.9	163309	88.2	55652	51.7
**Enrolled PLHIV started ART**	460,758	100.0	62,970	13.7	118,100	25.6	122,223	26.5	92,096	20.0	65,369	14.2
**Country**												
Tanzania	77,469	16.8	8,052	12.8	17,432	14.8	20,263	16.6	17,942	19.5	13,780	21.1
Mozambique	166,371	36.1	20,600	32.7	39,838	33.7	40,891	33.5	35,731	38.8	29,311	44.8
Kenya	132,307	28.7	19,199	30.5	34,135	28.9	39,635	32.4	23,691	25.7	15,647	23.9
Ethiopia	84,611	18.4	15,119	24.0	26,695	22.6	21,434	17.5	14,732	16.0	6,631	10.1
**Age at ART,** median (IQR)	34.0 (28.0–42.0)		36.0 (29.0–43.0)		35.0 (29.0–42.0)		35.0 (29.0–43.0)		34.0 (27.0–41.0)		31.0 (25.0–39.0)	
15–19 years	13,019	2.8	867	1.4	2,227	1.9	2,433	2.0	2,987	3.2	4,505	6.9
20–29 years	130,300	28.3	14,884	23.6	31,143	26.4	32,483	26.6	28,332	30.8	23,458	35.9
30–39 years	169,902	36.9	24,417	38.8	45,453	38.5	45,829	37.5	32,987	35.8	21,216	32.5
40–49 years	95,783	20.8	15,593	24.8	25,895	21.9	26,411	21.6	17,527	19.0	10,357	15.8
50+ years	51,754	11.2	7,209	11.4	13,382	11.3	15,067	12.3	10,263	11.1	5,833	8.9
**Male**	160,293	34.8	22,821	36.2	41,519	35.2	42,281	34.6	32,256	35.0	21,416	32.8
**Female**	300,465	65.2	40,149	63.8	76,581	64.8	79,942	65.4	59,840	65.0	43,953	67.2
Women pregnant at ART start	23,117	20.1	639	7.3	3,510	13.6	3,966	12.9	4,954	21.3	10,048	38.1
**CD4+, ART initiation,** median (IQR)	167 (80–261)		141 (65–215)		149 (72–227)		168 (81–254)		184 (88–287)		227 (109–350)	
CD4, women, median (IQR)	179 (90–275)		151 (73–225)		159 (81–237)		180 (92–266)		197 (100–298)		254 (128–393)	
CD4, men, median (IQR)	145 (65–236)		125 (55–200)		131 (59–208)		146 (65–235)		160 (72–262)		184 (84–300)	
**Missing CD4 count at ART initiation**	137,498	29.8	18,505	29.4	32,301	27.4	37,569	30.7	26,758	29.1	22,365	34.2
**WHO stage at ART initiation**												
Stage I	65,968	18.6	3,640	8.6	10,557	11.9	16,832	17.4	16,307	22.5	18,632	33.7
Stage II	87,798	24.7	7,694	18.1	20,044	22.6	26,748	27.7	20,220	27.9	13,092	23.7
Stage III	162,865	45.8	24,804	58.4	46,637	52.5	43,245	44.8	28,752	39.7	19,427	35.1
Stage IV	38,830	10.9	6,302	14.9	11,548	13.0	9,712	10.1	7,081	9.8	4,187	7.6
**Missing WHO stage at ART initiation**	105,297	22.9	20,530	32.6	29,314	24.8	25,686	21.0	19,736	21.4	10,031	15.3
**Days enrollment to ART initiation**	37 (10–173)		52 (9–231)		36 (9–221)		48 (14–236)		42 (15–159)		16 (0–55)	
**Never returned after ART start date**	39,996	8.7	4,558	7.2	9,231	7.8	8,215	6.7	7,614	8.3	10,378	15.9
**PLHIV in retention analyses**	432,817	93.9	62,784	99.7	117,174	99.2	119,793	98.0	85,619	93.0	47,447	72.6

Among the 802,348 ART-naïve PLHIV enrolled in care, 460,758 (57.4%) initiated treatment. There was an increase in the proportion of PLHIV starting ART at primary health facilities and those in rural areas (Table 1). The median age at ART initiation was 34 years (IQR: 28–42) and the majority (65.2%) were female. The proportion of women pregnant at ART initiation increased from 7.3% in 2005–2006 to 38.1% in 2013–2014 (p<0.0001). Median CD4+ increased from 141 cells/mm^3^ (IQR 65–215) in 2005–2006 to 227 cells/mm^3^ (IQR 109–350) in 2013–2014 (p<0.0001) (29.8% missing). Over time fewer PLHIV were WHO stage III/IV at ART initiation; 73.3% in 2005–2006 compared to 42.7% in 2013–2014 (p<0.0001). Median time from enrollment to ART initiation decreased over time from 52 days (IQR 9–231) to 16 days (IQR 0–55) from 2005–2006 to 2013–2014, respectively (p<0.0001) (Table 1). S3 Table has characteristics at ART initiation by country.

The cumulative risk of LTF for patients in care prior to ART initiation at 12, 24, and 36 months after enrollment in care was 33.5% (95%CI 33.4–33.6), 36.0% (95%CI 35.9–36.1) and 37.2% (95%CI 37.1–37.3), respectively (Table 2). Overall documented mortality at 12, 24, and 36 months after enrollment in care among PLHIV pre-ART was roughly 2% and decreased over time with highest mortality observed in Tanzania (5.0%, 95%CI 4.8–5.1), 36 months. Combined pre-ART attrition including LTF and death was 34.9% (95%CI 34.8–35.0, 37.5% (95%CI 37.4–37.6) and 38.7% (95%CI 38.6–38.8) at 12, 24 and 36 months, respectively, and decreased from 2005–2006 to 2013–2014 (p<0.0001). Women pregnant at enrollment and PLHIV 15–19 years of age had highest pre-ART attrition; 51.3% (95%CI 50.9–51.6) and 57.2% (95%CI 56.7–57.8) were not retained at 24 months, respectively (Table 2).

**Table 2:** Cumulative risk of pre-ART loss to follow-up, death and combined attrition for adults (> = 15 years) enrolled at ICAP-supported health facilities in Ethiopia, Kenya, Mozambique and Tanzania 2005–2014 (N = 792,319).

	Loss to follow-up			Death			Attrition (loss to follow-up and death)		
	12 months	24 months	36 months	12 months	24 months	36 months	12 months	24 months	36 months
	CR (95%CI)	CR (95%CI)	CR (95%CI)	CR (95%CI)	CR (95%CI)	CR (95%CI)	CR (95%CI)	CR (95%CI)	CR (95%CI)
**Overall**	33.5 (33.4–33.6)	36.0 (35.9–36.1)	37.22 (37.1–37.3)	2.2 (2.1–2.2)	2.4 (2.4–2.4)	2.5 (2.5–2.6)	34.9 (34.8–35.0)	37.5 (37.4–37.6)	38.7 (38.6–38.8)
**Enrollment year**	***p<0*.*0001***			***p<0*.*0001***			***p<0*.*0001***		
2005–2006	34.7 (34.4–34.9)	37.3 (37.1–37.6)	38.7 (38.5–39.0)	2.5 (2.4–2.6)	2.7 (2.6–2.8)	2.9 (2.8–3.0)	36.4 (36.1–36.6)	39.1 (38.8–39.3)	40.5 (40.2–40.7)
2007–2008	34.7 (34.5–34.9)	37.3 (37.1–37.5)	38.7 (38.5–38.9)	2.34 (2.3–2.4)	2.6 (2.6–2.7)	2.80 (2.7–2.9)	36.3 (36.1–36.5)	38.9 (38.7–39.2)	40.3 (40.1–40.5)
2009–2010	32.7 (32.5–32.9)	35.6 (35.4–35.8)	37.2 (37.0–37.4)	2.37 (2.3–2.5)	2.6 (2.6–2.7)	2.8 (2.7–2.9)	34.4 (34.2–34.6)	37.29 (37.1–37.5)	38.8 (38.6–39.0)
2011–2012	34.7 (34.5–35.0)	37.01 (36.8–37.3)	37.7 (37.4–37.9)	1.8 (1.7–1.9)	1.9 (1.8–2.0)	2.0 (1.9–2.1)	35.9 (35.7–36.2)	38.2 (38.0–38.4)	38.8 (38.6–39.1)
2013–2014	25.1 (24.7–25.5)	25.1 (24.7–25.5)	25.1 (24.7–25.5)	1.0 (0.9–1.1)	1.1 (1.0–1.2)	1.1 (1.0–1.2)	25.9 (25.5–26.3)	26.3 (25.9–26.7)	26.3 (25.9–26.7)
**Country**	***p<0*.*0001***			***p<0*.*0001***			***p<0*.*0001***		
Ethiopia	27.8 (27.6–28.1)	29.9 (29.7–30.2)	30.9 (30.6–31.1)	1.0 (0.9–1.0)	1.1 (1.0–1.1)	1.1 (1.1–1.2)	29.0 (28.8–29.6)	31.1 (30.9–31.4)	32.1 (31.9–32.4)
Kenya	26.7 (26.5–26.9)	28.9 (28.7–29.1)	30.0 (29.8–30.2)	2.4 (2.3–2.5)	2.6 (2.5–2.6)	2.66 (2.59–2.74)	28.4 (28.2–28.6)	30.6 (30.4–30.8)	31.7 (31.5–31.9)
Mozambique	43.4 (43.3–43.6)	46.4 (46.2–46.6)	47.9 (47.8–48.1)	1.7 (1.7–1.8)	1.9 (1.9–2.0)	2.1 (2.0–2.2)	44.4 (44.2–44.6)	47.4 (47.2–47.6)	48.9 (48.7–49.1)
Tanzania	25.0 (24.7–25.3)	27.3 (27.1–27.6)	28.5 (28.2–28.8)	4.3 (4.2–4.4)	4.8 (4.6–4.9)	5.0 (4.8–5.1)	28.2 (28.0–28.5)	30.7 (30.4–31.0)	31.9 (31.6–32.2)
**Sex**									
Men	32.4 (32.2–32.6)	34.5 (34.3–34.7)	35.5 (35.3–35.7)	2.9 (2.8–3.0)	3.2 (3.1–3.3)	3.3 (3.24–3.42)	34.4 (34.2–34.5)	36.5 (36.3–36.7)	37.5 (37.3–37.7)
Women (all)	34.0 (33.9–34.1)	36.7 (36.6–36.9)	38.1 (37.9–38.2)	1.8 (1.8–1.9)	2.0 (1.9–2.05)	2.1(2.1–2.2)	35.2 (35.1–35.3)	38.0 (37.8–38.1)	39.3 (39.2–39.4)
Pregnant women	47.6 (47.1–48.0)	50.9 (50.5–51.4)	52.7 (52.2–53.1)	0.8 (0.7–0.9)	0.9 (0.8–1.0)	1.0 (0.9–1.1)	48.0 (47.5–48.4)	51.3 (50.9–51.6)	53.1 (52.6–53.5)
**Age**	***p<0*.*0001***			***p<0*.*0001***			***p<0*.*0001***		
15–19	52.6 (52.1–53.2)	56.5 (55.9–57.0)	58.4 (57.8–58.9)	1.6 (1.5–1.8)	1.81 (1.6–2.0)	1.9 (1.7–2.2)	53.4 (52.9–54.0)	57.2 (56.7–57.8)	59.1 (58.6–59.6)
20–29	40.9(40.7–41.1)	44.0 (43.8–44.2)	45.5 (45.3–45.7)	1.7 (1.61–1.7)	1.9 (1.8–1.9)	2.0 (1.9–2.1)	41. (42.7–42.1)	45.0 (44.8–45.2)	59.1(58.6–59.6)
30–39	30.1 (29.9–30.2)	32.3 (32.1–32.5)	33.4 (33.2–33.6)	2.23 (2.2–2.3)	2.5 (2.4–2.5)	2.6 (2.5–2.6)	31.6 (31.4–31.8)	33.9 (33.7–34.1)	46.5 (46.3–46.7)
40–49	25.3 (25.0–25.5)	27.1 (26.9–27.8)	28.1 (27.8–28.3)	2.5 (2.5–2.6)	2.8 (2.7–2.9)	2.9 (2.8–3.0)	27.1 (26.9–27.4)	29.1 (28.8–29.3)	35.0 (34.8–35.2)
50+	25.1 (24.8–25.4)	27.1 (26.8–27.4)	28.0 (27.7–28.3)	3.1 (3.0–3.3)	3.4 (3.3–3.6)	3.6 (3.4–3.7)	27.4 (27.1–27.7)	29.5 (29.1–29.8)	30.0 (29.8–30.3)

*unadjusted sub-distributional hazards model testing difference in outcomes between all men and all women

**testing difference between men, non-pregnant women and pregnant women

LTF among PLHIV who initiated ART was 22.0% (95%CI 21.9–22.1), 29.5% (95%CI 29.4–29.7) and 35.4 (95%CI 35.3–35.6) at 12, 24, and 36 months, respectively (Table 3). LTF after ART initiation increased over time from 21.7% (95%CI 21.4–22.1) LTF by 24 months among PLHIV who enrolled in care 2005–2006 compared to 34.0% (95%CI 33.4–34.7) in 2013–2014 (p<0.0001). Loss to follow-up after ART initiation was highest in Mozambique where 45.2% (95%CI 44.9–45.5) of PLHIV who started treatment had been lost by 36 months. Men had higher attrition after ART start compared to women; at 24 months after ART initiation, 37.8% (95%CI 37.6–38.1) of men were not retained compared to 31.9% (95%CI 31.3–3.6) of women (p<0.0001). Pregnant women and the youngest PLHIV group had the highest attrition after ART initiation; at 24 months 40.8% (95%CI 40.1–41.6) of pregnant women and 47.4% (95%CI 46.4–48.4) of PLHIV 15–19 years were not retained (Table 3).

**Table 3:** Cumulative risk of loss to follow-up, death and combined attrition for adults (> = 15 years) after ART initiation enrolled at ICAP-supported health facilities in Ethiopia, Kenya, Mozambique and Tanzania 2005–2014 (N = 432,817).

	Loss to follow-up			Death			Attrition (loss to follow-up and death)		
	12 months	24 months	36 months	12 months	24 months	36 months	12 months	24 months	36 months
	CR (95%CI)	CR (95%CI)	CR (95%CI)	CR (95%CI)	CR (95%CI)	CR (95%CI)	CR (95%CI)	CR (95%CI)	CR (95%CI)
**Overall**	22.0 (21.9–22.1)	29.5 (29.4–29.7)	35.4 (35.3–35.6)	5.2 (5.1–5.3)	6.3 (6.2–6.4)	7.2 (7.1–7.3)	26.1 (26.0–26.2)	34.0 (33.8–34.1)	40.1 (40.0–40.3)
**Enrollment year**	***p<0*.*0001***			***p<0*.*0001***			***p<0*.*0001***		
2005–2006	16.7 (16.4–17.0)	21.7 (21.4–22.1)	27.2 (26.8–27.6)	7.4 (7.1–7.6)	8.7 (8.4–8.9)	9.6 (9.4–9.9)	22.8 (22.5–23.2)	28.6 (28.2–28.9)	34.3 (33.9–34.6)
2007–2008	21.2 (20.9–21.4)	28.0 (27.7–28.3)	33.5 (33.2–33.8)	5.7 (5.6–5.8)	6.8 (6.7–7.0)	7.8 (7.6–7.8)	25.73 (25.5–26.0)	33.0 (32.7–33.3)	38.7 (38.4–39.0)
2009–2010	20.0 (19.8–20.3)	29.3 (29.0–29.6)	36.4 (36.1–3.7)	4.8 (4.7–4.9)	5.9 (5.7–6.0)	6.7 (6.5–6.8)	23.90 (23.7–24.2)	33.44 (33.2–33.7)	40.7 (40.4–41.0)
2011–2012	26.0 (25.6–26.3)	35.05 (34.7–35.4)	40.9 (40.5–41.3)	4.4 (4.3–4.6)	5.2 (5.0–5.4)	6.0 (5.8–6.2)	29.3 (28.9–29.6)	33.4 (33.2–33.7)	40.7 (40.4–41.0)
2013–2014	29.0 (28.5–29.4)	34.0 (33.4–34.7)	N/A	3.3 (3.1–3.5)	4.5 (4.1–4.8)	N/A	31.4 (30.9–31.8)	37.0 (36.3–37.7)	N/A
**Country**	***p<0*.*0001***			***p<0*.*0001***			***p<0*.*0001***		
Ethiopia	18.1 (17.9–18.4)	24.4 (24.1–24.7)	28.8 (28.4–29.1)	5.4 (5.3–5.6)	6.3 (6.1–6.5)	6.8 (6.6–7.0)	22.6 (22.3–22.9)	29.2 (28.8–29.5)	33.6 (33.3–34.0)
Kenya	19.5 (19.2–19.7)	26.3 (26.1–26.6)	32.5 (32.3–32.8)	3.0 (2.9–3.1)	3.7 (3.6–3.8)	4.3 (4.2–4.4)	21.9 (21.7–22.2)	29.1 (28.9–29.4)	35.5 (35.2–35.8)
Mozambique	28.1 (27.9–28.4)	37.8 (37.6–38.1)	45.2 (44.9–45.5)	5.1 (5.0–5.3)	6.5 (6.3–6.6)	7.8 (7.6–8.0)	31.9 (31.7–32.1)	41.9 (41.6–42.2)	49.5 (49.2–49.8)
Tanzania	17.8 (17.5–18.1)	23.7 (23.3–24.0)	28.7 (28.0–29.1)	9.0 (8.8–9.2)	10.7 (10.5–11.0)	12.1 (11.8–12.4)	25.2 (24.9–25.6)	31.9 (31.6–32.3)	37.4 (37.0–37.8)
**Sex**	*p<0*.0001* *p<0*.0001**			*p<0*.0001* *p<0*.0001**			*p<0*.0001* *p<0*.0001**		
Men	24.1 (23.8–34.3)	31.9 (31.6–32.1)	38.1 (37.8–38.4)	7.13 (7.0–7.3)	8.6 (8.0–9.3)	9.9 (9.7–10.0)	29.5 (29.3–29.8)	37.8 (37.6–38.1)	44.3 (44.0–44.6)
Women (all)	20.9 (20.7–21.0)	28.2 (28.1–28.4)	33.99 (33.8–34.2)	4.19 (4.11–4.27)	5.1 (5.0–5.2)	5.8 (5.7–5.9)	24. (24.1–24.4)	31.9 (31.7–32.1)	37.8 (37.6–38.1)
Pregnant women	31.0 (30.3–31.7)	39.7 (38.9–40.5)	45.4 (44.5–46.3)	1.4 (1.2–1.6)	1.9 (1.7–2.2)	2.4 (2.1–2.8)	32.0 (31.3–32.6)	40.8 (40.1–41.6)	46.7 (45.9–47.6)
**Age**	***p<0*.*0001***			***p<0*.*0001***			***p<0*.*0001***		
15–19	34.8 (33.8–35.7)	44.0 (43.0–45.1)	50.5 (49.4–51.7)	4.6 (4.2–5.1)	5.9 (5.4–6.5)	7.1 (6.5–7.8)	37.8 (36.9–38.8)	47.4 (46.4–48.4)	54.1 (53.0–55.2)
20–29	26.5(26.3–26.8)	35.2 (34.9–35.5)	41.4 (41.141.7)	4.6 (4.5–4.8)	5.6 (5.5–5.8)	6.4 (6.3–6.6)	30.0 (29.7–30.2)	38.9 (38.6–39.2)	45.2 (44.9–45.5)
30–39	20.9 (20.7–21.1)	28.2 (27.9–28.4)	34.2 (34.0–34.5)	5.2 (5.1–5.3)	6.2 (6.0–6.3)	7.0 (6.8–7.1)	25.1 (24.8–25.3)	32.6 (32.4–32.9)	38.9 (38.6–39.1)
40–49	18.2 (18.0–18.5)	25.1 (24.8–25.5)	30.8 (30.4–31.1)	5.6 (5.4–5.8)	6.7 (6.5–6.9)	7.6 (7.4–7.8)	22.8 (22.6–23.1)	30.2 (29.9–30.5)	36.1 (35.7–36.4)
50+	18.3 (18.0–18.7)	25.0 (24.6–25.4)	30.4 (29.9–30.9)	6.1 (5.9–6.4)	7.6 (7.3–7.8)	8.9 (8.6–9.2)	23.4 (23.0–23.8)	30.7 (30.3–31.1)	36.6 (36.2–37.1)

*unadjusted Cox proportional hazards model testing difference in outcomes between all men and all women

**testing difference between men, non-pregnant women and pregnant women

Overall attrition (LTF and death) among all enrolled PLHIV regardless of ART status was 38.5 (95%CI 38.4–38.6) at 12 months, 44.4% (95%CI 44.3–44.5) at 24 months and 48.2% (95%CI 48.1–48.3) at 36 months (Table 4). Among all PLHIV enrolled 2005–2006, 48.0% (95%CI 47.7–48.3) were not retained in care at 36 months compared to 45.6% (95%CI 45.1–46.0) of those PLHIV enrolled 2013–2014 (p<0.0001). Attrition was higher among PLHIV with CD4+ >500 (39.9%, 95%CI 39.6–40.3, 24 months) compared to those with CD4<200 at enrollment (35.0%, 95%CI 34.8–35.2) (p<0.0001). Almost half of all men and women enrolled were not retained but among women who were pregnant at enrollment, 58.5 (95%CI 58.1–58.9) were not retained at 36 months (Table 4). Almost two-thirds (64.0%, 95%CI 63.5–64.6) of PLHIV 15–19 years of age and more than half (54.5%, 95%CI 54.3–54.7) of PLHIV 20–29 years were not retained at 36 months.

**Table 4:** Combined attrition for all adults (> = 15 years) in care regardless of ART initiation enrolled at ICAP-supported health facilities in Ethiopia, Kenya, Mozambique and Tanzania 2005–2014 (N = 792,319).

	Attrition (loss to follow-up and death)		
	12 months	24 months	36 months
	CR (95%CI)	CR (95%CI)	CR (95%CI)
**Overall**	38.5 (38.4–38.6)	44.4 (44.3–44.5)	48.2 (48.1–48.3)
**Enrollment year**	***p<0*.*0001***		
2005–2006	39.6 (39.3–39.9)	44.6 (44.3–44.9)	48.0 (47.7–48.3)
2007–2008	40.1 (39.9–40.3)	45.7 (45.5–45.9)	49.3 (49.1–49.5)
2009–2010	36.3 (36.1–36.5)	42.08 (41.87–42.28)	46.1 (45.9–46.3)
2011–2012	39.4 (39.2–39.7)	46.5 (46.2–46.7)	50.8 (50.6–51.1)
2013–2014	36.1 (35.6–36.5)	42.5 (42.1–42.9)	45.6 (45.1–46.0)
**CD4 count at enrollment**	***p<0*.*0001***		
<200	28.9 (28.7–29.1)	35.0 (34.8–35.2)	38.8 (38.6–39.0)
200–349	25.4 (25.0–25.6)	31.7 (31.4–32.0)	35.8 (35.4–36.1)
350–499	29.1 (28.8–29.5)	36.3 (35.9–36.7)	41.0 (40.6–41.4)
500+	32.4 (32.1–32.7)	39.9 (39.6–40.3)	44.7 (44.3–45.1)
Missing	48.5 (48.4–48.7)	53.7 (53.5–53.9)	57.2 (57.0–57.3)
**Country**	***p<0*.*0001***		
Ethiopia	31.2 (31.0–31.5)	38.2 (38.0–38.5)	42.0 (41.7–42.3)
Kenya	31.8 (31.6–32.0)	36.8 (36.6–37.0)	40.3 s(40.1–40.5)
Mozambique	47.7 (47.6–47.9)	54.0 (53.8–54.1)	58.1 (57.9–58.3)
Tanzania	33.9 (33.6–34.2)	39.3 (39.0–39.6)	42.6 (42.3–42.9)
**Sex**			
Men	40.3 (40.1–40.4)	46.6 (46.4–46.8)	50.5 (50.3–50.7)
Women (all)	37.6 (37.5–37.8)	43.3 (43.2–43.5)	47.1 (46.9–47.2)
Pregnant women	48.7 (48.3–49.1)	54.8 (54.4–55.2)	58.5 (58.1–58.9)
**Age at enrollment**	***p<0*.*0001***		
15–19	53.9 (53.3–54.4)	60.0 (59.4–60.5)	64.0 (63.5–64.6)
20–29	44.0 (43.8–44.2)	50.4 (50.3–50.6)	54.5 (54.3–54.7)
30–39	36.0 (35.8–36.2)	41.7 (41.5–41.9)	45.5 (45.3–45.6)
40–49	32.1 (31.9–32.4)	37.5 (37.2–37.7)	41.0 (40.7–41.3)
50+	32.4 (32.0–32.7)	37.9 (37.5–38.3)	41.51 (41.4–41.9)

## Conclusions

This analysis of almost one million PLHIV enrolled in care at 350 health facilities over ten years across four countries in Eastern and Southern Africa reflects the evolution of the HIV response over that decade, the progress made in expanding access to more populations with HIV, as well as the remaining challenges. By the end of the first decade of rapid ART expansion, a higher proportion of PLHIV entered care and started ART at primary health facilities and in rural areas, demonstrating successful decentralization of HIV services. Women continued to represent the majority of patients enrolled in care and starting ART, with a large increase in pregnant women starting ART over time. Median CD4+ at enrollment, while increasing over time, remained low at 289 cell/mm^3^ in 2013–2014. Attrition was high–roughly 20% of all PLHIV enrolled in care had no further recorded visits and almost 35% of PLHIV not yet on ART were LTF or had died within 12 months. While early attrition was lower among PLHIV after starting ART compared to those not on treatment, by 36 months estimates for attrition for patients not yet on ART and those on treatment were around 40%. In the latest period of observation, 2013–2014, pre-ART attrition had declined to 26% at 36 months but for PLHIV on ART, it had increased from 34% to 41%. Overall, regardless of ART status, almost half (48%) of PLHIV across the four countries were not retained at the facilities where they enrolled at 3 years after entry into HIV care with the highest attrition observed among pregnant women and young PLHIV.

A key finding from our analysis is the enormous increase in access to HIV care and treatment over time. Decentralization efforts have led to greater availability of HIV services at primary health clinics and in rural areas. Previous studies have demonstrated comparable health outcomes among PLHIV who receive care at lower level health facilities and lower rates of lost to follow-up among PLHIV initiating treatment at primary health clinics. [9–11] A study from Malawi found that in one district, expanding access to ART from the district hospital to primary health clinics decreased average travel distance from 7.3 to 4.7 kilometers and led to a 10% increase in visit attendance. [12] Decentralization has also been accompanied by task shifting, including expansion of nurse initiated ART (NIMART) and development of differentiated care models, such as community ART groups, that are continuing to expand access and make care more patient-oriented. [13–15]

Over the first decade of ART scale-up, women constituted the majority of PLHIV entering care and starting treatment. Our data reflect a dramatic increase in pregnant women initiating ART which could in part be due to improved reporting of pregnancy status at enrollment but mirrors changes in guidelines moving towards Option B+ (ART for all pregnant women). [16] We also found a high proportion of women enrolling in care who did not return after the first visit and alarmingly high LTF among pregnant women. Overall, 48% of pregnant women were lost before starting treatment and 31% of women who were pregnant at the time of ART initiation were lost to follow-up by 12 months. These data are consistent with previous reports of high loss to follow-up among pregnant women. [17,18] The follow-up period for this analysis ends at the time when many countries were expanding Option B+, however a recent systematic review of retention of pregnant and postpartum women in sub-Saharan Africa in the era of Option B+ by Knettel et al [19] found that only 76.4% (95%CI 69.0–83.1) were still in care at 12 months after enrollment. These results along with our findings from earlier periods underscore the urgent need to identify strategies to ensure that women remain in care and on treatment through pregnancy, breastfeeding period and thereafter. [20,21]

Perhaps one of the most alarming findings is the large number of patients LTF which is consistent with other studies, including our finding of the highest attrition among pregnant women and younger PLHIV. [8, 22–25] We also report a large proportion (20%) of patients who did not return after their first visit to the health facilities which few other retention analyses have documented. Tracing studies have provided important estimates of outcomes among PLHIV recorded as LTF in routine care settings. In a recent systematic review of outcomes of PLHIV on ART who were lost to follow-up in Africa, it was noted that 34% of patients successfully traced had died and 23.9% had transferred to another health facility, and that over time, in later cohorts, deaths appear to have declined while silent transfer have increased. [8] In a meta-analysis of data from nine tracing studies, it was noted that among PLHIV LTF after ART initiation, approximately 22% had died, 22% were alive but not on ART, and 15% had transferred to another clinic. [22] The authors also found that women and PLHIV with less advanced disease at ART initiation were more likely to have undocumented transfers. It is likely that some of the PLHIV identified as LTF in our analysis had undocumented transfer and may still be in care, nonetheless, it is still concerning that close to half of all patients enrolled in HIV care were not retained at three years. It is also possible that the introduction of “treat all” approaches (ART initiation at HIV diagnosis) as recommended by the WHO starting in 2015 will improve attrition; however there are few reports of long-term patient outcomes following the introduction of new guidelines from resource limited settings. While the data used for this analysis are several years old and may not reflect outcomes in the “treat all” era, our findings underscore the urgent need to identify drivers of LTF and to develop differentiated service delivery models that meet the needs and preferences of high-risk PLHIV. [14,15]

Encouragingly, our analysis showed improvement in disease stage among PLHIV at entry to HIV care over time reflecting expanded testing efforts and earlier engagement in care. While we saw fewer clinically advanced patients (WHO stage III/IV) at enrollment and increasing CD4+ counts at both entry and ART initiation, nonetheless, in 2013–2014, 36% of PLHIV enrolling in care across the four countries had CD4+ <200 cells/mm^3^. PHIA data highlight the continued challenges around early HIV diagnosis; in Tanzania (2016–2017) only 60% of PLHIV were aware of their HIV infection. [26] Even as attention has shifted toward ‘treat all’ with a focus on immediate ART initiation, it remains critical to identify and provide services targeted to the many PLHIV who continue to enter care and initiate ART with HIV. [27,28]

Strengths of this analysis include the very large sample size, multi-country cohorts, long duration of follow-up, which allowed for three-year retention estimates, and the timespan over the first decade of HIV treatment scale-up. In addition, our data cover the landscape of care settings from tertiary hospitals in urban areas to rural primary health clinics and are from multiple sub-national regions across four countries. These data are highly representative of where most PLHIV receive care in RLS, particularly in sub Saharan Africa, and reflect typical outcomes observed in real world settings. A key weakness is that our data are limited to information recorded in medical charts and are subject to significant amounts of documentation gaps. We also cannot distinguish between gaps in care and missing data, for instance we are unable to determine whether half of all PLHIV enrolled did not have a CD4+ test or whether their results were not recorded in the clinical chart. We also have limited death data drawn only from medical charts.

This analysis of almost one million PLHIV enrolled in HIV care and treatment during a critical decade in the HIV response offers insights from programmatic level supporting the enormous successes of the scale-up of HIV services as well as highlighting the challenges that still remain to be overcome.

## Supporting information

S1 Table National ART guidelines Ethiopia, Kenya, Mozambique, Tanzania and World Health Organization (WHO). (DOCX)

S2 Table Characteristics at enrollment among adults (> = 15 years) living with HIV enrolled in care at ICAP-supported facilities in Ethiopia, Kenya, Mozambique and Tanzania 2005–2014 by country (N = 884,328). (DOCX)

S3 Table Characteristics at ART initiation among adults (> = 15 years) living with HIV enrolled in care at ICAP-supported facilities in Ethiopia, Kenya, Mozambique and Tanzania 2005–2014 by year of enrollment (N = 460,758). (DOCX)
