# Supplementary material for: Expansion and scale-up of HIV care and treatment services in four countries over ten years
Source: PLoS One. 2020 Apr 16;15(4):e0231667. doi: 10.1371/journal.pone.0231667 (PMC7162457; doi:10.1371/journal.pone.0231667)
Supplement: S1 Table — (DOCX) [file pone.0231667.s001.docx]

**Supplemental Table 1.** National ART guidelines Ethiopia, Kenya, Mozambique, Tanzania and World Health Organization (WHO)

| **Years** | **Ethiopia** | **Kenya** | **Mozambique** | **Tanzania** | **WHO** |
| --- | --- | --- | --- | --- | --- |
| **2003** | - CD4<200 - WHO stage 4 | - CD4<200 - WHO stage 4 - CD4 200-350 & WHO stage 3 | - CD4<200 - WHO stage 4 - CD4 200-350 & WHO stage 3 | - CD4<200 - WHO stage 4 - CD4 200-350 & WHO stage 3 | - CD4<200 - WHO stage 4 - CD4 200-350 & WHO stage 3 |
| **2004** |  |  |  |  |  |
| **2005** |  |  |  |  |  |
| **2006** |  |  |  |  |  |
| **2007** |  |  |  |  |  |
| **2008** | - CD4<200 - WHO stage 4 - CD4 200-350 & WHO stage 3 |  |  |  |  |
| **2009** |  |  |  |  |  |
| **2010** |  |  | - CD4<250 - WHO stage 4 - CD4 250-350 & WHO stage 3 |  | - CD4<350   WHO stage 3 or 4 |
| **2011** |  | - CD4<350 - WHO stage 3 or 4 |  |  |  |
| **2012** |  |  | - CD4<350 - WHO stage 3 or 4 | - CD4<350 - WHO stage 3 or 4 |  |
| **2013** | - CD4<350 - WHO stage 4 |  | - CD4<350 - WHO stage 3 or 4 - All pregnant and breastfeeding women |  | - CD4<500 - WHO stage 3 or 4   All pregnant and breastfeeding women |
| **2014** | - CD4<350 - WHO stage 4 - All pregnant and breastfeeding women |  |  |  |  |

Ethiopia: in 2014 Ethiopia called for ART for all adults with CD4<500, WHO stage 3 or 4, anyone with TB, all pregnant and breastfeeding women and HIV-infected people with those with HIV-negative partners

Kenya: in June 2014 Kenya called for ART for all adults with CD4<500, all pregnant and breastfeeding women, all partners in sero-discordant couples, those with WHO stage 3 or 4 but not relevant for this analysis
Mozambique: March 2016 CD4 <500, WHO stage 3 or 4

Tanzania: Feb 2014 Tz launched Option B+ for all pregnant women and in 2015 called for ART for adults with CD4<500 or WHO stage 3 or 4, those with TB, key populations and those with HIV-negative partners
