# Supplementary material for: Expansion and scale-up of HIV care and treatment services in four countries over ten years
Source: PLoS One. 2020 Apr 16;15(4):e0231667. doi: 10.1371/journal.pone.0231667 (PMC7162457; doi:10.1371/journal.pone.0231667)
Supplement: S2 Table — (DOCX) [file pone.0231667.s002.docx]

|  | **Country** | | | | | | | | **All** | |
| --- | --- | --- | --- | --- | --- | --- | --- | --- | --- | --- |
|  | **Ethiopia** | | **Kenya** | | **Mozambique** | | **Tanzania** | |  |  |
|  | **N** | **%** | **N** | **%** | **N** | **%** | **N** | **%** | **N** | **%** |
|  | 152,052 | 17.2 | 234,648 | 26.5 | 365,809 | 41.4 | 131,819 | 14.9 | 884,328 | 100.0 |
| **Enrollment year** |  |  |  |  |  |  |  |  |  |  |
| 2005-2006 | 21,918 | 14.4 | 39,261 | 16.7 | 48,850 | 13.4 | 12,005 | 9.1 | 122,034 | 13.8 |
| 2007-2008 | 47,370 | 31.2 | 64,634 | 27.6 | 89,090 | 24.4 | 30,215 | 22.9 | 231,309 | 26.2 |
| 2009-2010 | 41,335 | 27.2 | 67,444 | 28.7 | 92,717 | 25.4 | 36,542 | 27.7 | 238,038 | 26.9 |
| 2011-2012 | 29,162 | 19.2 | 39,578 | 16.9 | 85,569 | 23.4 | 30,942 | 23.5 | 185,251 | 21.0 |
| 2013-2014 | 12,267 | 8.1 | 23,731 | 10.1 | 49,583 | 13.6 | 22,115 | 16.8 | 107,696 | 12.2 |
| **Facility type** |  |  |  |  |  |  |  |  |  |  |
| Primary | 28,794 | 18.9 | 61,738 | 26.3 | 174,681 | 47.8 | 32,153 | 24.4 | 297,366 | 33.6 |
| Secondary | 72,494 | 47.7 | 135,902 | 57.9 | 144,515 | 39.5 | 58,698 | 44.5 | 411,609 | 46.5 |
| Other | 50,764 | 33.4 | 37,008 | 15.8 | 46,613 | 12.7 | 40,968 | 31.1 | 175,353 | 19.8 |
| **Location** |  |  |  |  |  |  |  |  |  |  |
| Urban | 142,334 | 93.6 | 119,727 | 51.0 | 262,953 | 71.9 | 104,716 | 79.4 | 629,730 | 71.2 |
| Rural | 9,718 | 6.4 | 114,921 | 49.0 | 102,856 | 28.1 | 27,103 | 20.6 | 254,598 | 28.8 |
| **Number of health facilities reporting** | 73 | | 125 | | 67 | | 85 | | 350 | |
| **Age at enrolment,** median (IQR) | 30.0 (26.0-38.0) | | 34.0 (27.5-42.0) | | 30.0 (25.0-39.0) | | 35.3 (29.2-42.6) | | 32.0 (26.0-40.0) | |
| 15-19 years | 4,429 | 2.9 | 7,537 | 3.2 | 23,336 | 6.4 | 3,936 | 3.0 | 39,238 | 4.4 |
| 20-29 years | 55,978 | 36.8 | 71,007 | 30.3 | 142,521 | 39.0 | 33,532 | 25.4 | 303,038 | 34.3 |
| 30-39 years | 56,842 | 37.4 | 83,153 | 35.4 | 113,849 | 31.1 | 51,251 | 38.9 | 305,095 | 34.5 |
| 40-49 years | 23,990 | 15.8 | 45,606 | 19.4 | 56,427 | 15.4 | 28,689 | 21.8 | 154,712 | 17.5 |
| 50+ years | 10,813 | 7.1 | 27,345 | 11.7 | 29,676 | 8.1 | 14,411 | 10.9 | 82,245 | 9.3 |
| **Sex** |  |  |  |  |  |  |  |  |  |  |
| Female | 91,785 | 60.4 | 160,631 | 68.5 | 247,081 | 67.5 | 88,146 | 66.9 | 587,643 | 66.5 |
| Male | 60,267 | 39.6 | 74,017 | 31.5 | 118,728 | 32.5 | 43,673 | 33.1 | 296,685 | 33.6 |
| **Women pregnant at enrollment** | 8,030 | 8.7 | 6,392 | 8.0 | 40,237 | 16.4 | 12,311 | 18.5 | 66,970 | 16.7 |
| **Point of entry** |  |  |  |  |  |  |  |  |  |  |
| VCT | 46,076 | 30.3 | 78,354 | 33.4 | 105,058 | 28.7 | 63,121 | 47.9 | 292,609 | 33.1 |
| PMTCT | 5,307 | 3.5 | 19,469 | 8.3 | 46,576 | 12.7 | 14,720 | 11.2 | 86,072 | 9.7 |
| TB/HIV | 3,374 | 2.2 | 10,138 | 4.3 | 5,694 | 1.6 | 2,257 | 1.7 | 21,463 | 2.4 |
| Inpatient | 7,931 | 5.2 | 11,845 | 5.0 | 13,953 | 3.8 | 8,516 | 6.5 | 42,245 | 4.8 |
| Outpatient | 35,235 | 23.2 | 32,974 | 14.1 | 34,767 | 9.5 | 20,912 | 15.9 | 123,888 | 14.0 |
| Other | 30,045 | 19.8 | 56,036 | 23.9 | 133,125 | 36.4 | 13,159 | 10.0 | 232,365 | 26.3 |
| Unknown | 24,084 | 15.8 | 25,832 | 11.0 | 26,636 | 7.3 | 9,134 | 6.9 | 85,686 | 9.7 |
| **Transferred into care** | 12,558 | 8.3 | 13,803 | 5.9 | 16,402 | 99.99 | 12,770 | 9.7 | 55,533 | 14.0 |
| **CD4 at enrollment**, median (IQR) | 193 (91-357) | | 199 (73-383) | | 280 (135-471) | | 247 (104-453) | | 235 (103-426) | |
| <200 | 54,982 | 51.4 | 49,896 | 50.2 | 66,570 | 36.5 | 29,003 | 42.5 | 200,472 | 43.9 |
| 200-349 | 24,331 | 22.7 | 21,413 | 21.5 | 43,346 | 23.8 | 14,554 | 21.3 | 103,644 | 22.7 |
| 350-499 | 13,413 | 12.5 | 12,397 | 12.5 | 31,837 | 17.5 | 10,395 | 15.2 | 68,042 | 14.9 |
| 500+ | 14,260 | 13.3 | 15,748 | 15.8 | 40,555 | 22.2 | 14,278 | 20.9 | 84,841 | 18.6 |
| CD4, women, median (IQR) | 216 (104-391) | | 219 (85-416) | | 308 (156-502) | | 269 (119-479) | | 261 (120-460) | |
| CD4, men, median (IQR) | 163 (75-307) | | 164 (55-323) | | 229 (101-404) | | 207 (80-399) | | 192 (80-362) | |
| **Missing CD4 count at enrolment** | 49,056 | 29.6 | 140,303 | 57.6 | 184,188 | 50.2 | 63,589 | 48.2 | 427,329 | 48.3 |
| **WHO stage at enrollment** |  |  |  |  |  |  |  |  |  |  |
| Stage I | 42,904 | 30.4 | 54,288 | 29.2 | 87,593 | 34.1 | 35,380 | 29.0 | 220,165 | 31.2 |
| Stage II | 28,487 | 20.2 | 61,220 | 32.9 | 52,697 | 20.5 | 35,607 | 29.2 | 178,011 | 25.2 |
| Stage III | 57,670 | 40.8 | 64,548 | 34.7 | 97,906 | 38.1 | 33,912 | 27.8 | 254,036 | 36.0 |
| Stage IV | 12,178 | 8.6 | 5,988 | 3.2 | 18,906 | 7.4 | 16,971 | 13.9 | 54,043 | 7.7 |
| **Missing WHO stage at enrollment** | 10,813 | 7.1 | 48,604 | 20.7 | 108,707 | 29.7 | 9,949 | 7.5 | 178,073 | 20.1 |
| **Prior ART reported** | 39,225 | 25.8 | 10,123 | 8.8 | 554 | 0.2 | 15,750 | 13.7 | 65,652 | 8.9 |
| Women | 23,921 | 26.1 | 7,526 | 9.5 | 406 | 0.2 | 11,332 | 14.8 | 43,185 | 8.9 |
| Men | 15,304 | 25.4 | 2,597 | 7.1 | 148 | 0.1 | 4,418 | 11.5 | 22,467 | 9.0 |
| **On ART at time of enrollment** | 11,018 | 11.5 | 4,852 | 3.5 | 458 | 0.3 | 0 | 0.0 | 16,328 | 3.4 |
| **ART eligibility at enrollment by country guidelines*** | | |  |  |  |  |  |  |  |  |
| Eligible | 68,175 | 44.8 | 71,273 | 30.4 | 117,419 | 32.1 | 53,211 | 40.4 | 310,078 | 35.1 |
| Eligible started ART | 58,400 | 85.7 | 58,427 | 82.0 | 83,750 | 71.3 | 43,227 | 81.2 | 243,804 | 78.6 |
| Ineligible | 75,327 | 49.5 | 132,910 | 56.6 | 180,579 | 49.4 | 73,738 | 55.9 | 462,554 | 52.3 |
| Ineligible started ART | 35,317 | 46.9 | 69,867 | 52.6 | 67,676 | 37.5 | 32,366 | 43.9 | 205,226 | 44.4 |
| Indeterminate | 8,550 | 5.6 | 30,465 | 13.0 | 67,811 | 18.5 | 4,870 | 3.7 | 111,696 | 12.6 |
| Indeterminate started ART | 1,912 | 22.4 | 8,865 | 29.1 | 15,403 | 22.7 | 1,876 | 38.5 | 28,056 | 25.1 |
| **Never returned after enrollment** | 10,567 | 7.0 | 47,590 | 20.3 | 78,813 | 21.5 | 24,285 | 18.4 | 161,255 | 18.2 |
| Never returned, female | 6,559 | 62.1 | 32,174 | 67.6 | 54,039 | 68.6 | 16,195 | 66.7 | 108,967 | 67.6 |
| **Patients in retention analyses**** | 133,927 | 88.0 | 224,042 | 95.5 | 318,108 | 87.0 | 116,424 | 88.3 | 792,319 | 89.6 |

*Includes PLHIV on ART at enrollment in care**;** **Enrollment date >= 12 months prior to facility data end date and no reported prior ART

**Supplemental Table 2.** Characteristics at enrollment among adults (>=15 years) living with HIV enrolled in care at ICAP-supported facilities in Ethiopia, Kenya, Mozambique and Tanzania 2005-2014 by country (N=884,328)
