# Supplementary material for: Expansion and scale-up of HIV care and treatment services in four countries over ten years
Source: PLoS One. 2020 Apr 16;15(4):e0231667. doi: 10.1371/journal.pone.0231667 (PMC7162457; doi:10.1371/journal.pone.0231667)
Supplement: S3 Table — (DOCX) [file pone.0231667.s003.docx]

**Supplemental Table 3**. Characteristics at ART initiation among adults (>=15 years) living with HIV enrolled in care at ICAP-supported facilities in Ethiopia, Kenya, Mozambique and Tanzania 2005-2014 by year of enrollment (N=460,758)

|  | **Country** | | | | | | | | **All** | |
| --- | --- | --- | --- | --- | --- | --- | --- | --- | --- | --- |
|  | **Ethiopia** | | **Kenya** | | **Mozambique** | | **Tanzania** | |  |  |
|  | **N** | **%** | **N** | **%** | **N** | **%** | **N** | **%** | **N** | **%** |
|  | 84,611 | 18.40 | 132,307 | 28.7 | 166,371 | 36.1 | 77,469 | 16.8 | 460,758 | 100.0 |
| **Year of enrollment** |  |  |  |  |  |  |  |  |  |  |
| 2005-2006 | 15,119 | 17.9 | 19,199 | 14.5 | 20,600 | 12.4 | 8,052 | 10.4 | 62,970 | 13.7 |
| 2007-2008 | 26,695 | 31.6 | 34,135 | 25.8 | 39,838 | 23.9 | 17,432 | 22.5 | 118,100 | 25.6 |
| 2009-2010 | 21,434 | 25.3 | 39,635 | 30.0 | 40,891 | 24.6 | 20,263 | 26.2 | 122,223 | 26.5 |
| 2011-2012 | 14,732 | 17.4 | 23,691 | 17.9 | 35,731 | 21.5 | 17,942 | 23.2 | 92,096 | 20.0 |
| 2013-2014 | 6,631 | 7.8 | 15,647 | 11.8 | 29,311 | 17.6 | 13,780 | 17.8 | 65,369 | 14.2 |
| **Facility type** |  |  |  |  |  |  |  |  |  |  |
| Primary | 12,955 | 15.3 | 38,108 | 28.8 | 83,069 | 49.9 | 18,201 | 23.5 | 152,333 | 33.1 |
| Secondary | 41,757 | 49.4 | 74,236 | 56.1 | 64,665 | 38.9 | 33,099 | 42.7 | 213,757 | 46.4 |
| Others | 29,899 | 35.3 | 19,963 | 15.1 | 18,637 | 11.2 | 26,169 | 33.8 | 94,668 | 20.5 |
| **Location** |  |  |  |  |  |  |  |  |  |  |
| Urban | 79,807 | 94.3 | 66,668 | 50.4 | 120,343 | 72.3 | 61,417 | 79.3 | 328,235 | 71.2 |
| Rural | 4,804 | 5.7 | 65,639 | 49.6 | 46,028 | 27.7 | 16,052 | 20.7 | 132,523 | 28.8 |
| **Age at ART,** median (IQR) | 32.0 (27.0-39.0) | | 36.0 (30.0-44.0) | | 32.0 (26.0-40.0) | | 37.0 (31.0-44.0) | | 34.0 (28.0-42.0) | |
| 15-19 years | 1,851 | 2.2 | 2,334 | 1.8 | 7,745 | 4.7 | 1,089 | 1.4 | 13,019 | 2.8 |
| 20-29 years | 28,048 | 33.1 | 30,702 | 23.2 | 58,001 | 34.9 | 13,549 | 17.5 | 130,300 | 28.3 |
| 30-39 years | 33,849 | 40.0 | 49,461 | 37.4 | 55,487 | 33.4 | 31,105 | 40.2 | 169,902 | 36.9 |
| 40-49 years | 14,376 | 17.0 | 30,434 | 23.0 | 30,154 | 18.1 | 20,819 | 26.9 | 95,783 | 20.8 |
| 50+ years | 6,487 | 7.7 | 19,376 | 14.6 | 14,984 | 9.0 | 10,907 | 14.1 | 51,754 | 11.2 |
| **Sex** |  |  |  |  |  |  |  |  |  |  |
| Female | 50,117 | 59.2 | 89,416 | 67.6 | 109,815 | 66.0 | 51,117 | 66.0 | 300,465 | 65.2 |
| Male | 34,494 | 40.8 | 42,891 | 32.4 | 56,556 | 34.0 | 26,352 | 34.0 | 160,293 | 34.8 |
| **Women pregnant at ART** | 5,440 | 6.4 | 2,594 | 5.1 | 11,921 | 50.4 | 3,162 | 9.0 | 23,117 | 20.1 |
| **CD4 at ART initiation,** median (IQR) | 142 (74-214.0) | | 173 (75-271) | | 182 (92-283) | | 167 (76-273) | | 167 (80-261) | |
| <200 | 52,317 | 70.6 | 53,475 | 57.0 | 57,159 | 55.6 | 31,206 | 59.4 | 194,157 | 60.1 |
| 200-349 | 18,367 | 24.8 | 31,087 | 33.1 | 30,348 | 29.5 | 14,901 | 28.3 | 94,703 | 29.3 |
| 350-499 | 2,364 | 3.2 | 6,282 | 6.7 | 7,678 | 7.5 | 3,553 | 6.8 | 19,877 | 6.2 |
| 500+ | 1,011 | 1.4 | 2,991 | 3.2 | 7,602 | 7.4 | 2,919 | 5.6 | 14,523 | 4.5 |
| CD4, women, median (IQR) | 154 (83-225) | | 184 (85-281) | | 194 (104-299) | | 177 (86-283) | | 179 (90-275) | |
| CD4, men, median (IQR) | 125 (62-198) | | 151 (60-251) | | 159 (74-252) | | 147 (61-250) | | 145 (65-236) | |
| **Missing CD4 at ART initiation** | 10,552 | 12.5 | 38,472 | 29.1 | 63,584 | 38.2 | 24,890 | 32.1 | 137,498 | 29.8 |
| **WHO stage at ART initiation** |  |  |  |  |  |  |  |  |  |  |
| Stage I | 14,081 | 16.9 | 19,085 | 17.9 | 24,625 | 26.6 | 8,177 | 11.1 | 65,968 | 18.6 |
| Stage II | 17,762 | 21.4 | 34,946 | 32.9 | 18,563 | 20.1 | 16,527 | 22.5 | 87,798 | 24.7 |
| Stage III | 42,326 | 50.9 | 48,018 | 45.1 | 40,680 | 44.0 | 31,841 | 43.4 | 162,865 | 45.8 |
| Stage IV | 9,014 | 10.8 | 4,344 | 4.1 | 8,623 | 9.3 | 16,849 | 23.0 | 38,830 | 10.9 |
| **Missing WHO stage at ART initiation** | 1,428 | 1.7 | 25,914 | 19.6 | 73,880 | 44.4 | 4,075 | 5.3 | 105,297 | 22.9 |
| **Days from enrollment to ART initiation**, median (IQR) | 21 (7-129) | | 59 (17-287) | | 39 (13-133) | | 26 (4-126) | | 37 (10-173) | |
| **Never returned after ART start date** | 683 | 0.8 | 15,074 | 11.4 | 17,310 | 10.4 | 6,929 | 8.9 | 39,996 | 8.7 |
| **Patients in retention analysis*** | 81,078 | 95.8 | 129,751 | 98.1 | 151,180 | 90.9 | 70,808 | 91.4 | 432,817 | 93.9 |

*ART start date >= 6 months prior to facility data end date
